# Preliminary evidence for obesity-associated insulin resistance in adolescents without elevations of inflammatory cytokines

**DOI:** 10.1186/1758-5996-4-26

**Published:** 2012-06-08

**Authors:** Jessica I Cohen, Lawrence Maayan, Antonio Convit

**Affiliations:** 1 Department of Psychiatry, New York University School of Medicine, 145 East 32nd St, New York, NY, 10016, USA; 2 Department of Medicine, New York University School of Medicine, 145 East 32nd St, New York, NY, 10016, USA; 3 Nathan Kline Institute for Psychiatric Research, 140 Old Orangeburg Rd, Orangeburg, NY, 10962, USA

**Keywords:** Insulin resistance, Cytokines, Adipokines, Adolescents, Obesity

## Abstract

**Background:**

To ascertain whether the associations between obesity, inflammation, and insulin resistance established in human adult studies are found among adolescents.

**Methods:**

We contrasted 36 obese and 24 lean youth on fasting glucose, insulin levels, lipid profile, hemoglobin A1C, markers of hepatic function, white blood cell count, C-reactive protein (CRP) and fibrinogen levels. The cytokines IL-6, TNF-α, IFN-γ, IL-10 and IL-4 and the adipokines leptin, resistin, and adiponectin were also compared between the two groups. The fasting glucose and insulin values were used to estimate the degree of insulin resistance with the homeostatic model assessment of insulin resistance (HOMA-IR). T-tests and correlations were run to examine group differences and associations between groups. In addition, regression analyses were used to ascertain whether the markers of inflammation were predictive of the degree of insulin resistance.

**Results:**

Although obese adolescents had clear evidence of insulin resistance, only CRP, fibrinogen and leptin were elevated; there were no group differences in pro- or anti-inflammatory cytokines nor adiponectin and resistin. Anthropometric measures of obesity and level of insulin resistance were highly correlated to the acute phase reactants CRP and fibrinogen; however, the degree of insulin resistance was not predicted by the pro- or anti-inflammatory cytokine markers. Obese adolescents had higher white blood cell counts. In addition they had higher circulating alanine aminotransferase concentrations and lower circulating albumin and total protein than lean adolescents, possibly as a result of hepatocyte damage from fatty liver.

**Conclusion:**

Unlike rodent or adult studies, we found that wide-spread systemic inflammation is not necessarily associated with insulin resistance among adolescents. This finding does not support the current paradigm that the associations between obesity and insulin resistance are, to a significant degree, mediated by low grade systemic inflammation. These data support the need for further adolescent studies to explore these associations.

## Introduction

Obesity is considered a low-grade chronic inflammatory disease that may contribute to the development of insulin resistance [1]. Obese adults and adolescents are at higher risk for developing type 2 diabetes, cardiovascular disease, non-alcoholic fatty liver disease (NAFLD) and several forms of cancer [2-5]. Previous research suggests that mediators of low grade chronic inflammation, such as cytokines and acute phase reactants, contribute to the development of these co-morbid conditions [6,7]. In the adult human, the very high co-occurrence of obesity, inflammation and insulin resistance bolsters the hypothesis that obesity-associated inflammation may lead to insulin resistance [8,9]. However, there is not complete consensus on this matter [10]. It is important to improve our understanding of the pathophysiology involved in the progression from obesity to insulin resistance, particularly in youth where obesity and insulin resistance may still be dissociated.

Over the last two decades, our view of adipose tissue was transformed from that of an “inert” storage tissue to an endocrine organ that secretes a number of proteins, such as adiponectin, resistin, and leptin [11-14]. Adiponectin is anti-inflammatory, while resistin is pro-inflammatory, and their imbalance can result in low grade inflammation [15,16]. Leptin, another adipokine that increases with adiposity, is an important satiety signal, and obese individuals may become resistant to leptin resulting in greater production and secretion of this protein [8,17]. Both leptin and resistin are correlated with insulin resistance and are considered key mediators linking insulin resistance with hepatic steatosis [18].

As adipocytes store larger lipid droplets and increase in size, the physical stress on the blood vessels that supply them increases leading to a compromised endothelial lining [19]. This facilitates macrophage infiltration, which in turn increases cytokine production [6]. Another possible cause of increased cytokine expression is damage to specific organs, for example the liver. Hepatic steatosis is an imbalance in triglyceride acquisition and removal, resulting in the storage of excess triglycerides by hepatocytes. This can result in the increased production of inflammatory cytokines, such as tumor necrosis factor (TNF)-α, by resident macrophages [3]. Acute phase reactants produced in the liver, such as C-reactive protein (CRP) and fibrinogen, are also elevated in obese adults and are implicated in the development of cardiovascular, kidney disease and diabetes [20-22].

The pathophysiology of inflammation among obese children and adolescents is less well-developed, and no clear consensus exists in the pediatric literature [23,24]. Most pediatric studies show consistent increases in CRP, but not increases in interleukin (IL)-6 and TNFα nor decreases in IL-10 or IL-4. These are important issues to consider given the large variability in the degree of insulin resistance among obese adolescents [25,26].

We hypothesized that the associations between key biomarkers of obesity and insulin resistance in adolescents may exist at an organ system level but perhaps not systemically, thus resulting in the inconsistent results reported in this age group. To test this hypothesis we assessed markers of the immune system, liver markers, pro- and anti-inflammatory cytokines, adipokines, and acute phase reactants in a group of lean and obese adolescents. Based on clear fasting hyperinsulinemia among our obese adolescents, suggesting significant levels of insulin resistance, we also ascertained whether obesity/insulin resistance is associated with systemic changes in adipokines and elevations in acute phase reactants and inflammatory cytokines.

## Materials and methods

### Participants

Sixty adolescents, 24 lean and 36 obese, matched on age, gender, school grade, ethnicity, and socioeconomic status (Table 1) were studied. The subjects were consecutive cases evaluated as part of an NIH-sponsored study at the Brain, Obesity, and Diabetes Laboratory, Department of Psychiatry, New York University School of Medicine. The study was approved by the local IRB. All of the participants (and, if they were under 18 years of age, their parent) signed informed consent and received compensation for their time and inconvenience. Participants were screened to rule out exclusionary preexisting medical and psychiatric conditions. Hypertension, dyslipidemia, and insulin resistance were allowed. None of the study subjects were receiving any psychotropic medications. The original participant pool contained 117 adolescents. Of the 117 adolescents, 19 were excluded because they had either CRP > 10 mg/L, which may be indicative of an acute inflammation or infection [27], or had missing CRP values. Additionally, 18 possible participants were excluded because they were receiving anti- inflammatory or β-agonist medications and 10 had a history of inflammatory illnesses (e.g., asthma). Lastly, 10 participants did not have stored plasma available for measurement of cytokine and adipokine concentrations. The 57 excluded and 60 evaluated did not differ on age, race, gender, or BMI.

**Table 1:** Group differences in demographic and biological parameters

	**Lean**	**Obese**	**p-value**
Gender (% female)	58.3	55.6	0.832
Race/Ethnicity			χ^2^ = 1.02
White (%)	33.3	38.9	
Hispanic (%)	25.0	27.8	
African American (%)	25.0	25.0	
Asian (%)	16.7	8.3	
Age (yrs)	17.2 ± 0.4	17.5 ± 0.3	0.396
Grade	11.2 ± 0.4	11.4 ± 0.2	0.708
BMI (kg/m^2^)	21.5 ± 0.5	37.4 ± 1.2	0.000**
Waist Circumference (cm)	76.8 ± 1.2	112.2 ± 2.8	0.000**
Waist:Height	0.45 ± 0.01	0.66 ± 0.01	0.000**
Waist:Hip	0.85 ± 0.01	0.94 ± 0.01	0.000**
Glucose (mg/dL)	73.5 ± 1.4	77.3 ± 1.5	0.083
Insulin (IU/mL)	7.4 ± 0.6	18.4 ± 2.0	0.000**
HOMA-IR	1.3 ± 0.1	3.5 ± 0.4	0.000**
Hemoglobin A1C (%)	5.1 ± 0.1	5.4 ± 0.1	0.000**
Total cholesterol (mg/dL)	160.5 ± 5.9	164.1 ± 5.3	0.662
LDL (mg/dL)	91.5 ± 5.1	102.6 ± 4.7	0.125
HDL (mg/dL)	55.0 ± 2.5	43.7 ± 1.5	0.000**
Triglycerides (mg/dL)	70.6 ± 5.6	89.4 ± 7.2	0.064
Systolic BP (mmHg)	101.3 ± 1.9	115.3 ± 2.0	0.000**
Diastolic BP (mmHg)	63.2 ± 1.7	68.0 ± 1.5	0.036*

*Denotes a p-value of >0.05; **denotes a p-value of ≤0.010.

Body mass index (BMI); homeostatic model assessment of insulin resistance (HOMA-IR); low density lipoprotein (LDL); high density lipoprotein (HDL); blood pressure (BP).

### Blood collection

Following a 10–12 hour fast, blood was collected from all participants at 09:00 hours in a standardized fashion. Standard blood tests, such as CBC with differential, comprehensive metabolic profile including glucose and insulin levels, lipid profile, hemoglobin A1C, CRP, fibrinogen levels, white blood cell (WBC) count and markers of liver function were performed at the NYU Medical Center Clinical Laboratories. In addition to the blood samples used for the clinical labs, samples were collected in EDTA tubes, placed immediately on ice, spun at 4°C at 1,700 RPM for 10 minutes, and the plasma was stored at −80°C for future cytokine and adipokine assays, which were performed at the NYU Clinical Translational Science Institute core laboratory.

### Estimation of insulin resistance

As an estimate of insulin resistance, fasting glucose and insulin levels were used to compute the homeostatic model assessment of insulin resistance (HOMA-IR). HOMA-IR has been validated against the hyperinsulinemic-euglycemic clamp [28]. HOMA-IR values above 3.16 are considered indicative of insulin resistance in adolescence, whereas those under 1.99 suggest an absence of insulin resistance [29].

### Circulating cytokine and adipokine measurements

Plasma samples were thawed on ice. ELISAs using Multiplex plates (Millipore, Billerica, MA) were performed. The plates were pre-coated with antibodies to the proteins of interest and plasma samples were loaded onto the plates per the manufacturer’s instructions. Plates were read on a Luminex plate reader (Austin, TX). Protein concentrations were determined by comparing the unknown sample concentrations to those of standard curves.

### Statistical analysis

Data were analyzed using SPSS for Windows version 19.0 (SPSS, Inc., Chicago, IL). The Shapiro-Wilk test was used to test data normality. Independent t-tests were used to investigate group differences in demographics, metabolic parameters including HOMA-IR and fasting insulin levels, liver parameters, white cell counts, adipokines, cytokines and acute phase proteins. Exploratory analyses were conducted using the Pearson’s or Spearman’s correlation to investigate whether markers of adiposity, insulin resistance, liver injury and immune responses were associated with circulating CRP and fibrinogen levels. We used linear and hierarchical regressions to account for potential confounders, such as gender, ethnicity and blood pressure. To formally test whether levels of the inflammatory markers accounted for the degree of insulin resistance, we used linear regressions with HOMA-IR (or fasting insulin level) as the dependent variable, and after accounting for possible confounds (age and gender), we evaluated the contribution of the different cytokines to the variance in these dependent variables.

## Results

### Demographic and endocrine data

The participants were, on average, approximately 17 years of age with an 11^th^ grade education (Table 1). As expected the obese group had significantly higher HOMA-IR, fasting insulin, and hemoglobin A1C values compared to the lean group. Although all subjects had fasting glucose levels in the normal range, the obese group tended towards higher fasting glucose levels (Table 1). The average BMI of the lean group was 21.5 kg/m^2^ and the average BMI for the obese group was 37.4 kg/m^2^. Not surprisingly, the obese group had larger waist circumference and larger waist-to-height and waist-to-hip ratios than the lean group. Lastly, the obese group also had lower HDL concentrations and higher blood pressure than the lean group (Table 1). Although the differences in triglyceride concentrations did not reach statistical significance, there was a statistical trend for increased triglyceride concentration in the obese group (Table 1).

The liver panel revealed no group differences in aspartate aminotransferase (AST), alkaline phosphatase, or bilirubin. However, as indicated on Table 2, obese adolescents had higher circulating concentrations of alanine aminotransferase (ALT). Both circulating total protein and albumin were higher in the lean group and the obese group had a higher WBC value than the lean group (Table 2).

**Table 2:** Hepatic markers and white blood cell (WBC) count

	**Lean**	**Obese**	**p-value**
ALT (U/L)	23.9 ± 1.2	42.3 ± 5.5	0.002**
AST (U/L)	29.3 ± 2.5	31.0 ± 2.4	0.647
Alkaline Phosphatase (mg/dL)	91.4 ± 6.1	92.8 ± 4.7	0.855
Total Bilirubin (U/L)	0.5 ± 0.1	0.4 ± 0.0	0.416
Total Protein (gm/dL)	7.8 ± 0.5	7.5 ± 0.4	0.010**
Albumin (mg/dL)	4.7 ± 0.3	4.3 ± 0.3	0.000**
WBC (10^3^/mL)	5.5 ± 0.8	6.8 ± 1.8	0.000**

**denotes a p-value of ≤0.010.

Alanine aminotrasferase (ALT); aspartate aminotransferase (AST); white blood cell (WBC).

### Differences in cytokine, adipokine, and acute phase reactant concentrations between lean and obese adolescents

There were no group differences in the concentration of either the pro-inflammatory cytokines IL-6, TNFα, and IFN-γ (Table 3) or the anti-inflammatory cytokines IL-10 and IL-4 (Table 3). There were also no group differences in adiponectin and resistin. However, relative to lean adolescents, obese adolescents had nearly a 4-fold higher leptin concentration (Table 3). Obese adolescents also had a significant elevation (nearly 7-fold higher) in circulating CRP levels compared to the lean adolescents (Table 3). Please note that all adolescents with CRP levels greater than 10 mg/dl were excluded from the sample [27]. All of the lean adolescents had CRP levels under 1 mg/dl. The obese group also had significantly higher fibrinogen levels (about a 14 % elevation) compared to the lean group (Table 3).

**Table 3:** Circulating cytokine, adipokine and acute phase reactant concentrations

	**Lean**	**Obese**	**p-value**
TNFα (pg/ml)	3.4 ± 2.1	3.5 ± 2.0	0.900
IL-6 (pg/ml)	8.1 ± 6.8	9.3 ± 8.4	0.588
IFN-γ (pg/ml)	14.1 ± 12.8	11.4 ± 8.0	0.321
IL-4 (pg/ml)	77.4 ± 54.8	79.5 ± 49.1	0.877
IL-10 (pg/ml)	22.5 ± 21.4	26.3 ± 46.3	0.715
Leptin (ng/ml)	10.9 ± 11.1	38.1 ± 25.6	0.000**
Adiponectin (ng/ml)	766.8 ± 165.9	112.0 ± 233.1	0.652
Resistin (pg/ml)	102.8 ± 65.3	119.2 ± 117.3	0.538
*Acute phase reactants*			
CRP (mg/L)	0.5 ± 0.4	3.3 ± 2.2	0.000**
Fibrinogen (mg/L)	278.2 ± 33.3	327.9 ± 54.4	0.000**

**denotes a p-value of ≤0.010.

Tumor necrosis factor (TNF); interferon (IFN); interleukin (IL); C-reactive protein (CRP).

There were strong positive associations between CRP and fibrinogen and BMI, waist circumference, waist-to-height ratio and HOMA-IR values (Table 4). CRP concentrations were positively associated with ALT and WBC and negatively associated with total protein and albumin (Table 5). Fibrinogen was positively associated with ALT and WBC (Table 5).

**Table 4:** Associations between acute phase reactants and indicators of obesity and insulin resistance

**CRP**	**Correlation Coefficient**	**p-value**	**Fibrinogen**	**Correlation Coefficient**	**p-value**
BMI	0.748	0.000**	BMI	0.490	0.000**
Waist Circumference	0.785	0.000**	Waist Circumference	0.532	0.000**
Waist:Height	0.774	0.000**	Waist:Height	0.550	0.000**
HOMA-IR	0.520	0.000**	HOMA-IR	0.475	0.001**

**denotes a p-value of ≤0.010.

Body mass index (BMI); homeostatic model assessment of insulin resistance (HOMA-IR).

**Table 5:** Associations between acute phase reactants and hepatic markers and white blood cell (WBC) count

	**Correlation Coefficient with CRP**	**p-value**	**Correlation Coefficient with Fibrinogen**	**p-value**
ALT	0.535	0.000**	0.464	0.001**
Total Protein	−0.264	0.042*	−0.066	0.654
Albumin	−0.377	0.003**	−0.250	0.087
WBC	0.447	0.000**	0.461	0.001**

*denotes a p-value of <0.05; **denotes a p-value of ≤0.010.

Alanine aminotrasferase (ALT); white blood cell (WBC).

None of the pro- or anti-inflammatory cytokines levels were predictive of fasting insulin levels or HOMA-IR scores for all subjects combined or either group alone.

## Discussion

We found clear elevations in the acute phase reactants CRP and fibrinogen, as well as in insulin resistance among obese adolescents. However, we found no differences in pro- or anti-inflammatory cytokine expression between obese and lean adolescents, nor did we find any association between cytokine levels and our measures of insulin resistance. This could be partly an issue of detection, since cytokine concentrations are generally orders of magnitude lower than what is seen with acute phase reactants. However, given that we had detectable levels of all cytokines, just no differences between lean and obese, and that this is in agreement with other studies in youth, our results suggest that the association between cytokines and insulin resistance may differ in youth and adults. Importantly, we had a clear difference in the level of insulin resistance between lean and obese groups, as well as clear associations between insulin resistance and measures of obesity and of acute phase reactants.

While circulating pro-inflammatory cytokines are, in the majority of studies, elevated in adult obesity [8-10], the literature on inflammatory cytokines in adolescents is quite mixed [23,24]. Tam et al. [30] suggests that ethnicity and gender may contribute to differences in cytokine expression; however, in this study neither gender nor ethnicity accounted for the lack of cytokine differences by BMI group. Our negative results may be in part due to low statistical power from our relatively small sample size. However, another possible explanation for the differences between adult and adolescent studies is that adolescents may have been obese for a relatively short time. Increases in inflammatory cytokine concentrations are, in part, the result of macrophage infiltration into the adipose tissue [6,31,32], and it is possible that more time is required for this infiltration to occur. The current data is consistent with others showing that increases in CRP and fibrinogen precede increases in cytokine expression in the circulation [30]. Furthermore, unlike cytokines which are primarily produced by activated macrophages, CRP and fibrinogen expression can be elevated through other mechanisms such as activation of the complement system, a component of the innate immune response [33]. We found that the obese group had higher WBC count, suggesting activation of the innate immune response.

Although differences in circulating levels of cytokines were not observed, it is possible that there was increased cytokine expression in specific organs like the liver. Obesity and elevated fasting glucose levels are risk factors for the development of NAFLD [18]. Lipolysis of adipose tissue, *de novo* hepatic lipogenesis, and diet are the main sources of hepatic triglycerides [3]. The accumulation of triglycerides in the cytosol of hepatocytes leads to cell damage and increased production of inflammatory cytokines [18]. In this study, obese adolescents had higher circulating levels of ALT, an enzyme that is released into the circulation specifically by injured hepatocytes [34]. The elevated ALT, higher triglycerides, and greater central obesity present in our obese group may suggest the presence of hepatic inflammation, which in turn may contribute to the elevated acute phase reactants seen among them. Additionally, our obese adolescents had lower protein and albumin in their circulation, which may also suggest hepatic injury or compromise [35].

In adults, the current paradigm is that obesity-mediated insulin resistance contributes to impaired endothelial cell function. There are several mechanisms that may lead to compromised endothelium. One possible cause of these impairments may be the accumulation of advanced glycation end-products, a byproduct of glycation and oxidative stress [36]. These impairments in endothelial function allow macrophages to extravasate from blood vessels into adipose tissue [8,37]. It is these infiltrating macrophages, rather than the stromovascular cells or the adipocytes themselves, that produce inflammatory cytokines [37]. This infiltration changes the environment of the adipose tissue making it more pro-inflammatory [38]. Furthermore, insulin resistance itself leads to activation of pro-inflammatory M1 macrophages in the adipose tissue leading to increased cytokine and chemokine production and recruitment of foreign macrophages into the tissue [18]. Longitudinal studies of youth are needed to elucidate if and how the adipose tissue environment changes in response to obesity.

Rodent studies suggest that obesity-associated low grade chronic inflammation is critical for the development of insulin resistance [39,40]. The c-Jun N-terminal kinase pathway is central in the cascade leading from inflammation to insulin resistance. These relationships are not well understood, and most human studies in adults are cross-sectional and only establish a co-occurrence of inflammation and insulin resistance, but cannot inform which occurs first [8,9]. The few existent prospective human studies use non-naturalistic experimental diet paradigms that cause rapid weight gain, but also hyperglycemia, and are therefore not optimal to elucidate the physiological mechanism behind gradual weight gain leading to insulin resistance [32]. In contrast to adult studies, where obesity is most frequently associated with insulin resistance, adolescents can be obese without having overt insulin resistance. For this reason, adolescents provide a more straightforward opportunity to investigate the causal relationship between inflammation and insulin resistance in the human. Our data clearly suggests that among obese adolescents insulin resistance may precede the elevation of systemic cytokines and some adipokines.

The adipokines adiponectin and resistin were not different across our lean and obese adolescent groups, whereas leptin was significantly higher in the obese group. Unlike adiponectin and resistin, leptin is a satiety signal [41]. Leptin expression can be increased by gastric hormones like ghrelin [42] and as a compensatory mechanism for the leptin resistance that is frequently observed in obesity [43]. Also, unlike adiponectin and resistin, which are only produced by adipocytes, leptin is also produced by gastric epithelium cells and glands in the gastric fundus [44]. Lastly, adiponectin and resistin are more sensitive to changes in inflammatory homeostasis. Increases in inflammation can decrease adiponectin and increase resistin; however, leptin expression is not as sensitive to these biological changes [8]. These relationships are clearly captured in our data, where we showed elevations in the acute phase reactants and leptin but not in inflammatory cytokines nor adiponectin and resistin.

Our study has some notable limitations. We do not know how long our subjects were obese, but given their young age, variability is likely somewhat limited. Additionally, as a measure of insulin resistance we used HOMA-IR, which although highly correlated to the results of dynamic assessments of insulin function such as the hyperinsulinemic-euglycemic clamp, remains a single point static measure. Future studies should utilize dynamic assessments of insulin function. Our study is strengthened by the examination of several pro- and anti- inflammatory cytokines and adipokines, using well-established methods. The cytokines and adipokines measured in this study are strongly associated with obesity and insulin resistance in adults [9]. All of the samples we assayed for cytokines and adipokines were measured simultaneously, which limited variability inherent in different kits. This also obviated the need for repeated freezing and thawing of the samples. An additional strength of this study was our naturalistic study design. Unlike other human studies, our obese adolescents with insulin resistance were carefully matched to lean subjects and both groups had normal fasting glucose levels, which allowed us to ascertain these associations during the natural development of obesity and insulin resistance, independent of rapid weight gain and hyperglycemia from experimental diet interventions [32].

In the future more studies are needed to elucidate the pathogenesis leading to insulin resistance. Follow up studies should include measures of ceremide concentration [45], TNFα receptor [46] and markers of polycystic ovary syndrome. It is still unknown how long excess adiposity needs to be present before macrophage infiltration begins. Furthermore, if contrary to the widely held belief, a systemic increase in cytokine concentration does not necessarily precede insulin resistance, more work needs to be done in the adolescent human to identify other possible mechanisms that contribute to insulin resistance.

## Competing interests

The authors have no competing interests to disclose.

## Authors’ contributions

JIC carried out and interpreted the data analysis, organized data into tables, wrote the manuscript and gave final approval. LM was involved with the interpretation of the data the drafting of the manuscript and gave final approval. AC was involved in the conceptual design of the study, drafting of the manuscript and gave final approval. All authors read and approved the final manuscript.
